# Supplementary material for: Gnas Promoter Hypermethylation in the Basolateral Amygdala Regulates Reconsolidation of Morphine Reward Memory in Rats
Source: Genes (Basel). 2022 Mar 21;13(3):553. doi: 10.3390/genes13030553 (PMC8950747; doi:10.3390/genes13030553)
Supplement: Supplementary file 1 [file genes-13-00553-s001.zip › Supplementary Materials.pdf]

## Supplemental information:

**Figure S1. Schematic representation of the injection sites in the basolateral amygdala (BLA) (A), central amygdala (CeA) (B), nucleus accumbens (NAc) shell (C), and NAc core (D)**. Numbers beside the sections indicate anteroposterior distance from the bregma in millimetres. Data are reconstructed from Paxinos and Watson 2005 (Paxinos & Watson, 2005).

**Figure S2. DNA methylation of *Sox10* at 1 h after retrieval.** (A) The DNA methylation level of *Sox10* as evaluated by reduced-representation bisulphite sequencing. Differentially methylated region (DMR) located in the promoter region of Sox10 was identified (difference in DNA methylation level > 0.2, p = 0.010233, CpG number ⩾ 5). (B) The DNA methylation level of *Sox10* evaluated by bisulphite PCR validation (p > 0.05, Student's t-test, two samples, each consisting of the pooled tissues of six rats).

**Figure S3. DNA methylation of *Pik3r1* at 1 h after memory retrieval.** (A) The DNA methylation level of Pik3r1 evaluated by reduced-representation bisulphite sequencing. Differentially methylated region (DMR) located in the promoter region of Pik3r1 was identified (difference in DNA methylation level > 0.2, p =

0.010744, CpG number ⩾ 5). (B) The DNA methylation level of Pik3r1 evaluated by bisulphite PCR validation ($p > 0.05$, Student's t-test, two samples, each consisting of the pooled tissues of six rats).

Paxinos, G., & Watson, C. (2005). The rat brain in stereotaxic coordinates. 5 ed. *Amsterdam: Elsevier Academic Press.*
