# Supplementary figures and images for: Gnas Promoter Hypermethylation in the Basolateral Amygdala Regulates Reconsolidation of Morphine Reward Memory in Rats
Source: Genes (Basel). 2022 Mar 21;13(3):553. doi: 10.3390/genes13030553 (PMC8950747; doi:10.3390/genes13030553)

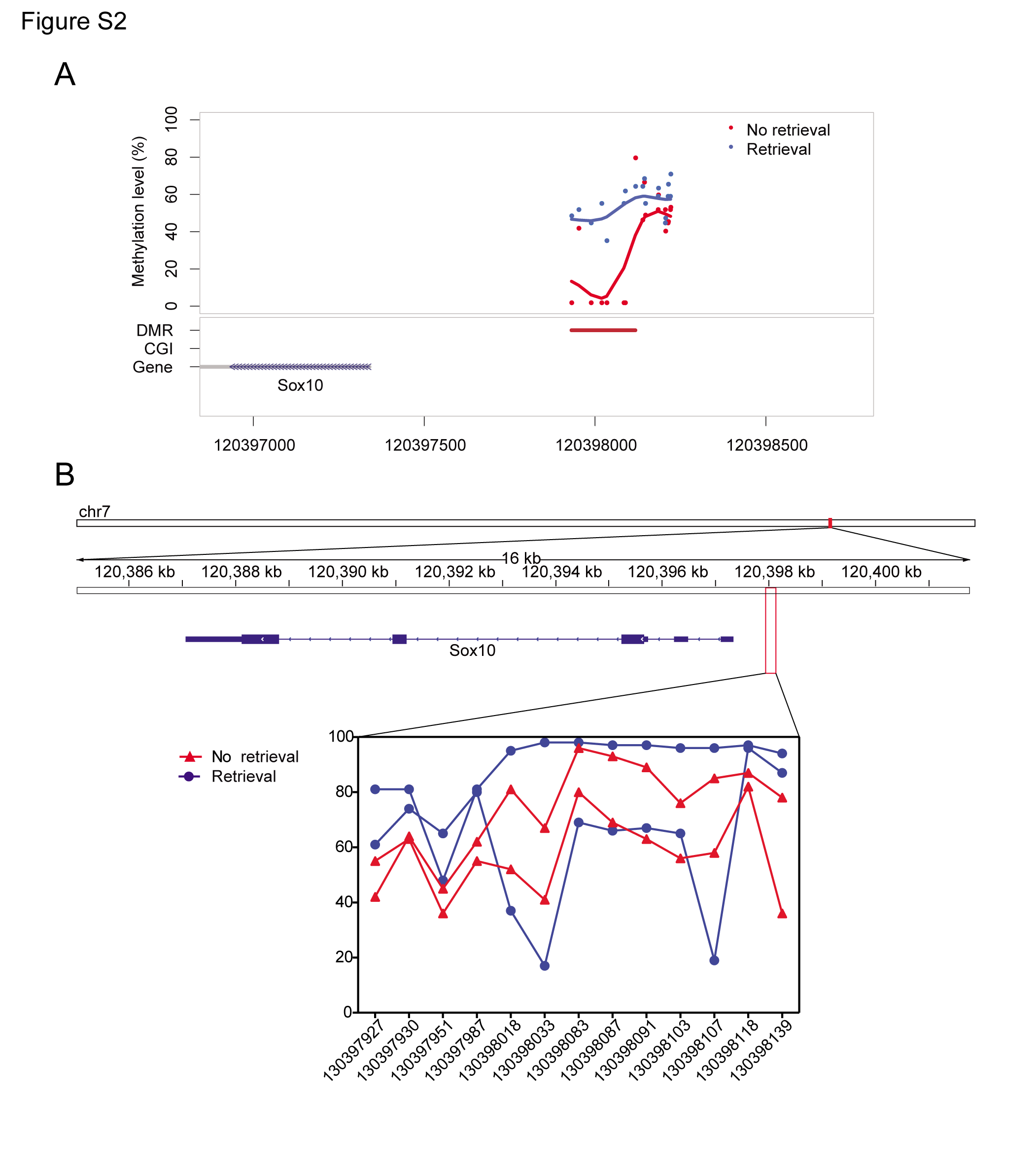

Supplement: Supplementary file 1 [file genes-13-00553-s001.zip › Supplementary Figure S2.tif]

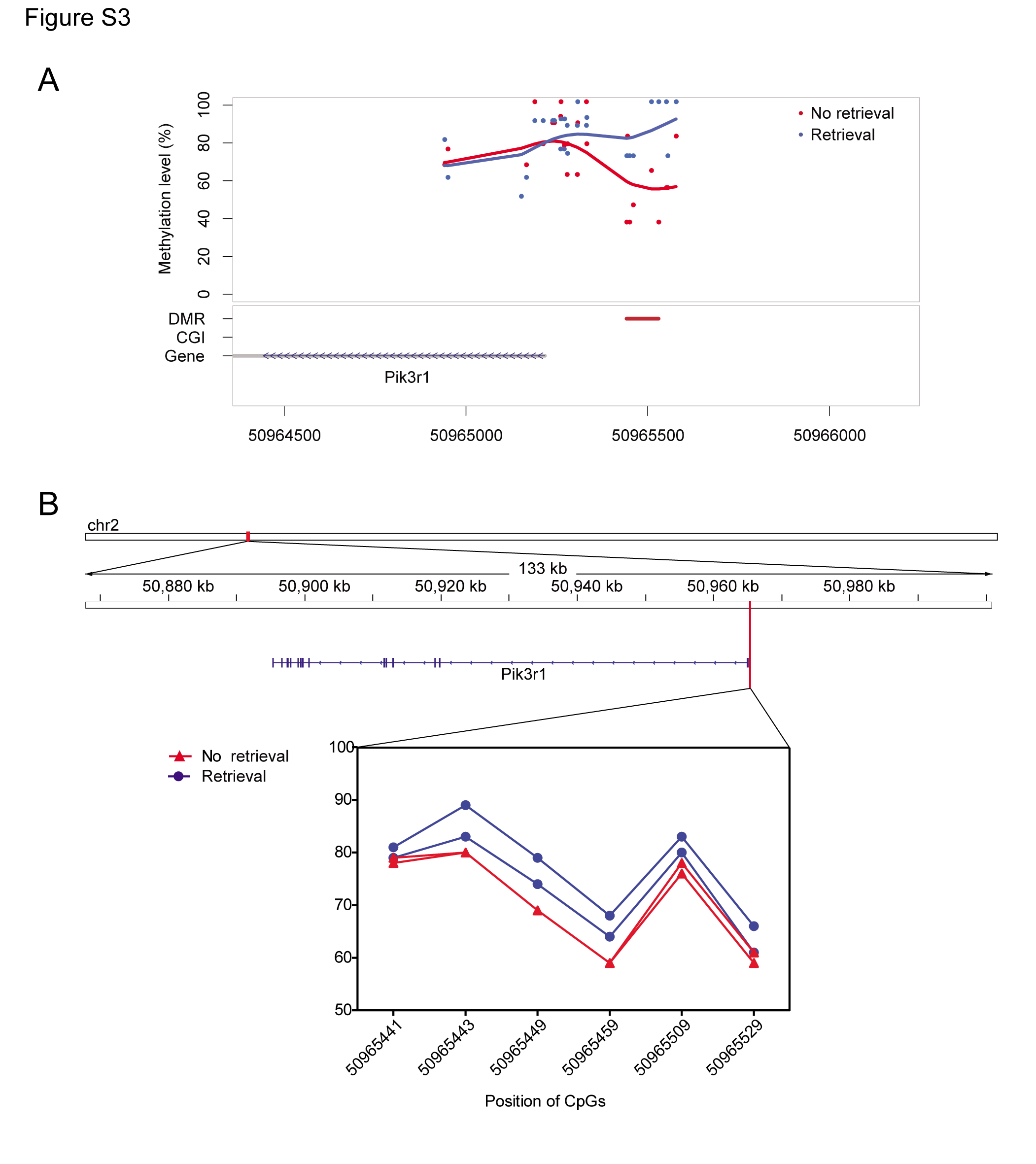

Supplement: Supplementary file 1 [file genes-13-00553-s001.zip › Supplementary Figure S3.tif]
